# Proposal for a Pharmacogenetic Decision Algorithm

**DOI:** 10.7759/cureus.1289

**Published:** 2017-05-30

**Authors:** Saeed K Alzghari, Lori Blakeney, Kerry Anne Rambaran

**Affiliations:** 1 Gulfstream Genomics, Gulfstream Diagnostics; 2 Department of Pharmacy Practice, Texas Tech University Health Sciences Center School of Pharmacy

**Keywords:** pharmacogenetics, biotechnology, cytochrome p450, pharmacokinetics, pharmacodynamics

## Abstract

Personalized medicine is playing an ever-increasing role in patient care. Over the past decade, awareness of the role of pharmacogenetics and its benefits is leading to its growing acceptance among providers. Though providers are using pharmacogenetics in practice, the decision-making process of when to use this tool can be ambiguous. Herein, we propose an algorithm to help guide providers on when to use pharmacogenetics for patient care.

## Editorial

The advent of personalized medicine is revolutionizing healthcare. Pharmacogenetics is the study of drug responses in relation to specific genetic polymorphisms [1]. The cost of performing pharmacogenetic testing is falling, leading to increased opportunities for patient-centered care [2]. Adverse drug effects and treatment failure are two key reasons as to why pharmacogenetics is gaining acceptance among clinicians as a tool for building assessments and subsequent treatment plans for patients [3-5].

**Figure 1:**
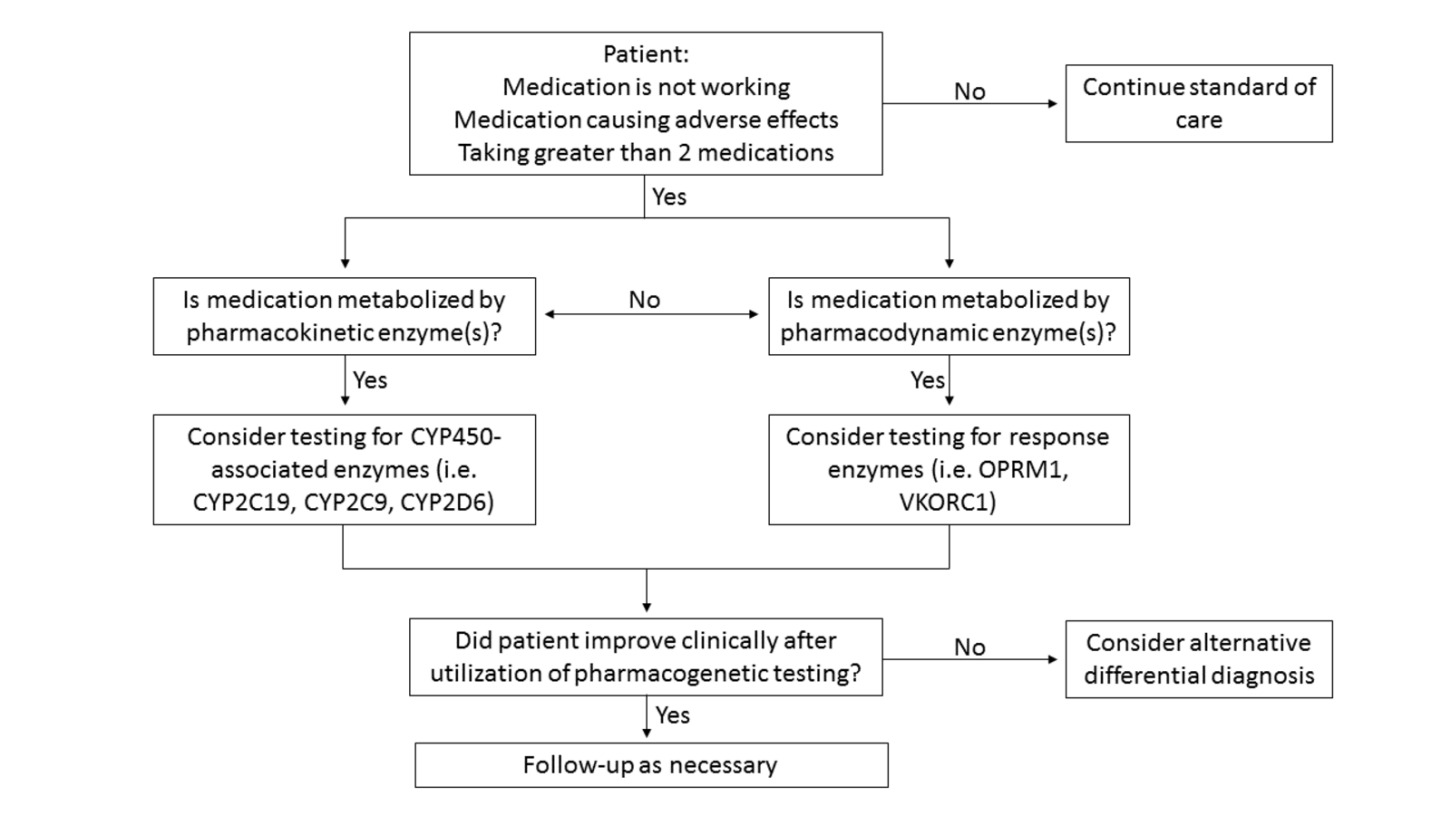
Pharmacogenetic decision algorithm

In considering utilizing pharmacogenetics, a clinician needs to have a clear reason as to why a pharmacogenetic test is ordered. We propose an algorithm describing when to perform a pharmacogenetic test (Figure 1). If a patient’s medication is not therapeutically effective, is causing a number of adverse effects, or if a patient is taking two or more medications, a clinician can consider performing a pharmacogenetic test on their patient. A clinician must also consider whether the medications in question metabolize through pharmacokinetic enzymes (i.e., what the body does to the drug) or pharmacodynamic enzymes (i.e., what the drug does to the body) to affect response. If pharmacokinetic enzymes are involved, testing for cytochrome P450 enzymes such as CYP2C19, CYP2C9, CYP2D6, and others can determine whether patients might have a poor, intermediate, normal, or rapid metabolizer status. Furthermore, it is important to consider if the medication(s) a patient may be on are cytochrome P450 inhibitors (i.e., ritonavir) or inducers (i.e., rifampin) that could lead to drug-drug interactions. If pharmacodynamic enzymes are involved, testing for enzymes such as the mu-opioid receptor 1 (OPRM1) or vitamin K epoxide reductase subunit 1 (VKORC1) can determine responses to certain medications. If the patient improved clinically after a medication dose change or after taking alternative medication based on pharmacogenetic testing, then follow-up with the patient as necessary. If the patient did not improve, a clinician should consider alternative differential diagnoses. 

In our experience with clinicians considering performing pharmacogenetic testing, the decision of when to perform the actual test comes into question. The algorithm we propose is a simple tool for clinicians to reference in making their decision. One very important point is that the subjective (i.e., past medical history, reported response to medications) and objective (i.e., clinical lab panels) information of a patient needs to be considered before deciding on a pharmacogenetic test. Incomplete or missing medical information may limit the utility of a pharmacogenetic test since the entire patient picture may not be clearly in view.

In conclusion, pharmacogenetic testing may be of great benefit to the right patient requiring this test. We want our algorithm to help in this clinical decision-making process and believe this is a tool to guide clinicians wanting to perform this type of test.
